# Sex-Specific Association of Blood Pressure Categories With All-Cause Mortality: The Rural Chinese Cohort Study

**DOI:** 10.5888/pcd17.190131

**Published:** 2020-01-30

**Authors:** Leilei Liu, Bingyuan Wang, Xincan Liu, Yongcheng Ren, Yang Zhao, Dechen Liu, Junmei Zhou, Xuejiao Liu, Dongdong Zhang, Xu Chen, Cheng Cheng, Feiyan Liu, Qionggui Zhou, Jianxin Li, Jie Cao, Jichun Chen, Jianfeng Huang, Ming Zhang, Dongsheng Hu

**Affiliations:** 1 Department of Epidemiology and Health Statistics, College of Public Health, Zhengzhou University, Zhengzhou, Henan, People’s Republic of China; 2 Department of Preventive Medicine, Shenzhen University Health Science Center, Shenzhen, Guangdong, People’s Republic of China; 3 Department of Cardiology, The First Affiliated Hospital of Henan University of Chinese Medicine, Zhengzhou, Henan, People’s Republic of China; 4 Study Team of Shenzhen’s Sanming Project, The Affiliated Luohu Hospital of Shenzhen University Health Science Center, Shenzhen, Guangdong, People’s Republic of China; 5 Department of Epidemiology, Fuwai Hospital, National Center for Cardiovascular Diseases, Chinese Academy of Medical Sciences and Peking Union Medical College, Beijing, People’s Republic of China

## Abstract

**Introduction:**

The relationship between blood pressure categories and all-cause mortality has not been fully addressed in cohort studies, especially in the general Chinese population. Our study aimed to assess the sex-specific association of systolic blood pressure (SBP), diastolic blood pressure (DBP), and 2017 United States hypertension guidelines with all-cause mortality in China.

**Methods:**

We conducted a prospective study of 13,760 rural Chinese adults aged 18 or older (41.1% men). Mean age overall was 49.4, 51.0 for men, and 48.3 for women. We analyzed the blood pressure–mortality relationship by using restricted cubic splines and Cox proportional-hazards regression analysis, estimating hazard ratios (HRs) and 95% confidence intervals (CIs).

**Results:**

During a mean follow-up of 5.95 years, 710 people died (60.3% men) from any cause. We found a U-shaped SBP–mortality or DBP–mortality relationship for both sexes. Mortality risk was increased for men with SBP 120–139 mm Hg (adjusted HR [aHR], 1.42; 95% CI, 1.10–1.82) or ≥140 mm Hg (aHR, 2.05; 95% CI, 1.54–2.72), and for DBP ≥90 mm Hg (aHR, 1.53; 95% CI, 1.10–2.13) as compared with SBP 100–119 mm Hg or DBP 70–79 mm Hg. Mortality risk also was increased for men with blood pressure status defined according to 2017 US hypertension guidelines as elevated, SBP 120–129 and DBP >80 mm Hg (aHR 1.48; 95% CI,1.11–1.98); stage 1 hypertension, SBP/DBP 130–139/80–89 mm Hg (aHR 1.53; CI, 1.19–1.97); and stage 2 hypertension, SBP/DBP ≥140/90 mm Hg (aHR 1.83; CI, 1.33–2.51). No significant relationship was observed for women.

**Conclusion:**

Elevated blood pressure and stages 1 and 2 hypertension were positively associated with all-cause mortality for men but not women in rural China.

**Summary**

**What is already known on this topic?**

Increased risk of all-cause mortality is associated with nonoptimal blood pressure. However, studies of the relationship between blood pressure categories and all-cause mortality in the Chinese adult population are limited, and sex-specific studies of these associations are not available. Furthermore, the relationship between the 2017 US hypertension guidelines and all-cause mortality in China is unclear.

**What is added by this report?**

Results of our prospective study, in a rural Chinese population, showed that risk of all-cause mortality with hypertension based on the 2017 US hypertension guidelines was increased for men but not women.

**What are the implications for public health practice?**

Treatment for hypertension should rely on clinicians’ cautious judgment about whether and when to start treatment. In addition, antihypertension goals may need to be individualized for people with different characteristics.

## Introduction

Nonoptimal blood pressure, which caused over 10 million deaths worldwide in 2016, is the leading cause of global mortality (1,2). Epidemiologic evidence has indicated a U- or J-shaped association of systolic blood pressure (SBP) or diastolic blood pressure (DBP) with all-cause mortality (2–4). Previous cohort studies investigated the SBP–mortality or DBP–mortality relationship in the general Chinese population; however, study populations were mainly coalminers, urban women, or people aged 65 or older (4–8). Also, to our knowledge, no other general population–based studies have been made of the relationship between blood pressure categories and all-cause mortality in China except for 2 cohort studies, 1 of adults aged 40 or older and 1 of urban women (6,9). In addition, studies of Chinese adults that consider the potential sex difference in the blood pressure–mortality relationship are lacking (9,10).

The 2017 US hypertension guidelines recommend maintaining blood pressure levels of SBP <130 mm Hg and DBP <80 mm Hg in the general population (11); the guidelines lowered recommended blood pressure levels compared with previous US and Chinese recommendations (12–14). Only 1 study, in Singapore — a population that is not representative of mainland China — evaluated the association between blood pressure categories and mortality on the basis of 2017 US hypertension guidelines (15).

We conducted a prospective cohort study in a rural Chinese population to elucidate the sex-specific association of baseline SBP and DBP with all-cause mortality and also examined the association between the 2017 US hypertension guidelines and all-cause mortality in China.

## Methods

### Study participants

**Figure 1:**
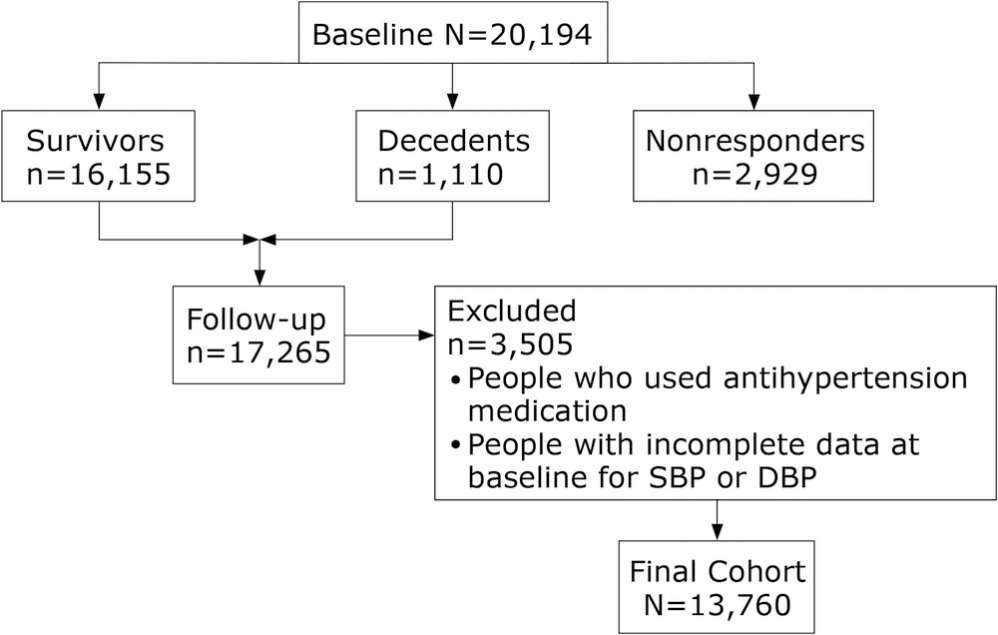
Flow diagram of participant selection, Sex-Specific Association of Blood Pressure Categories With All-Cause Mortality: The Rural Chinese Cohort Study, 2007–­2014. Abbreviations: DBP, diastolic blood pressure; SBP, systolic blood pressure.

We conducted a prospective cohort study of 20,194 participants aged 18 or older who were recruited from rural areas of Henan Province in China for baseline examination (July–August 2007 and July–August 2008). During a mean follow-up of 5.95 years, 17,265 study participants were re-investigated, from July through August 2013 and July through October 2014, with a response rate of 85.5%. Details of the cohort were described previously (16). After excluding people who used antihypertension medication or whose data were incomplete for SBP or DBP at baseline examination (n = 3,505), the final cohort consisted of 13,760 eligible study participants (41.1% men, n = 5,661) (Figure 1).

All study participants gave their informed consent for inclusion before the start of the study, and the study was approved by the ethics committee of Zhengzhou University.

**Baseline measurements.** During face-to-face interviews, trained interviewers administered a standard questionnaire to collect sociodemographic information (sex, age, marital status, income, and education level), behavioral measures (smoking, drinking, and physical activity), and medical history for all study participants. Details regarding variables and the questionnaire for this cohort have been published (16).

Smoking was classified as currently smoking and/or having smoked 100 or more cigarettes during their lifetime (17). Alcohol drinking was defined as having consumed alcohol 12 or more times in the past year. Physical activity level was classified as low, moderate, or high according to the International Physical Activity Questionnaire (18).

Weight and height were measured twice according to a standard protocol, weight to the nearest 0.1 kg and height to the nearest 0.1 cm, with study participants wearing light clothing but no shoes. An average of the 2 measurements was used in our study. Body mass index (BMI) was calculated as weight (kg) divided by height (m) squared (19).

Blood pressure was measured 3 times at 30-second intervals in the right arm after 5 minutes of rest with participants in a seated position by using an electronic sphygmomanometer (HEM-770AFuzzy, Omron, Kyoto, Japan) according to the American Heart Association standardized protocol. The average value of the 3 measurements was used for analyses (20).

Overnight fasting blood samples from each participant were collected in vacuum tubes to assess levels of total cholesterol, triglycerides, high-density lipoprotein cholesterol (HDL-C), and fasting plasma glucose by using an automated biochemical analyzer (Hitachi 7080, Tokyo, Japan) with reagents from Wako Pure Chemical Industries (Osaka, Japan). Low-density lipoprotein cholesterol (LDL-C) level was calculated by using the Freidwald formula (21).

**Blood pressure categories.** We classified SBP as <100 mm Hg, 100 to 119 mm Hg (reference), 120 to 139 mm Hg, and ≥140 mm Hg (7,22), and DBP as <70 mm Hg, 70 to 79 mm Hg (reference), 80 to 89 mm Hg, and ≥90 mm Hg (23). To better characterize actual blood pressure levels and to evaluate the sex-specific association of blood pressure with all-cause mortality, we further classified study participants into 4 groups according to the 2017 US hypertension guidelines (11): normal blood pressure (SBP <120 mm Hg and DBP <80 mm Hg), elevated blood pressure (SBP 120–129 mm Hg and DBP <80 mm Hg), stage 1 hypertension (SBP 130–139 mm Hg or DBP 80–89 mm Hg), and stage 2 hypertension (SBP ≥140 mm Hg or DBP ≥90 mm Hg).

**Mortality ascertainment during follow-up.** We collected data on time and cause of death by face-to-face interviews with relatives, local village physicians, or other health care providers on the basis of a standard questionnaire; we checked corresponding information for death with the local Center for Disease Control and Prevention. All-cause mortality was defined according to codes A00-Z99 of the International Classification of Diseases, 10th Revision (24).

### Statistical analyses

Baseline data for study participants were median (interquartile range) because of skewed distribution for continuous variables and number (percentage) for categorical variables. Differences in baseline characteristics by mortality status were compared by using the Mann–Whitney U test for continuous variables and χ^2^ test for categorical variables. Person years of follow-up for each study participant were computed by date of death or follow-up minus baseline examination date.

We used restricted cubic splines to test a possible dose–response association of SBP or DBP with all-cause mortality, by sex, at baseline examination as a continuous variable. In addition, we used Cox proportional-hazards regression analysis, calculating hazard ratios (HRs) and 95% confidence intervals (CIs). To avoid potential bias and confirm primary findings, we performed additional sensitivity analyses because of a rapid decrease in strength of the blood pressure–mortality association during the early follow-up period (25); study participants who had died during the first 2 years of follow-up (n = 143) were excluded from analyses. We further excluded participants with myocardial infarction, heart failure, stroke, diabetes mellitus, or cancer at baseline (n = 1,314) because these diseases could have affected blood pressure and thus the blood pressure–mortality relationship (2,26). All analyses were adjusted at baseline examination for age; marital status; mean individual income (monthly); education level; smoking; drinking; physical activity; BMI; total cholesterol; triglycerides; HDL-C; LDL-C; fasting plasma glucose; family history of hypertension, diabetes mellitus, or hyperlipidemia; and use of hypoglycemic and lipid-lowering medications.

All data were analyzed by using SAS version 9.4 (SAS Institute, Inc), and figures were plotted by using Stata 12 software (Stata Corp LLC). Statistical significance was set at a 2-tailed *P* < .05.

## Results

**Baseline characteristics of study participants.** Among the 13,760 study participants at baseline examination, 41.1% (n = 5,661) were men, and the median age was 49 (interquartile range, 40–59) (Table). During a mean follow-up of 5.95 years (81,856 person years), 710 deaths occurred. Of the 710 decedents, 428 (60.3%) were men, 679 (95.6%) had less than a high school diploma, 540 (76.1%) were married or cohabiting; 264 (37.2%) were smokers, 69 (9.7%) drank alcohol, 684 (96.3%) had a mean individual monthly income below 500 Chinese yuan (CNY), and 364 (51.3%) had a low physical activity level. The median SBP of decedents was 126.3 (interquartile range [IQR], 115.3–141.7); DBP, 76.3 (IQR, 69.0–84.3); total cholesterol, 4.5 (IQR, 3.9–5.1); LDL-C, 2.6 (IQR 2.2–3.1); fasting plasma glucose, 5.4 (IQR, 5.0–6.0); and lower BMI and HDL-C level (all *P* < .05). The median age of decedents was 65.0 (IQR 57.0–73.0). Appendix Table 1 shows the baseline characteristics of people included and excluded.

**Table:** Baseline Characteristics of Study Participants by Mortality Status, the Rural Chinese Cohort Study, 2007–2014^a^

Variable	Total (N = 13,760)	Survivors (n = 13,050)	Decedents (n = 710)	*P *Value
**Men**	5,661 (41.1)	5,233 (40.1)	428 (60.3)	<.001
**Age, y, median (IQR)**	49.0 (40.0–59.0)	49.0 (40.0–58.0)	65.0 (57.0–73.0)	<.001
**Married or cohabitating**	12,460 (90.6)	11,920 (91.3)	540 (76.1)	<.001
**Mean individual income, monthly, CNY**				
<500	12,792 (93.2)	12,108 (92.8)	684 (96.3)	.001
500–1,000	744 (5.4)	722 (5.5)	22 (3.1)	
≥1,000	191 (1.4)	189 (1.4)	2 (0.3)	
**Education level high school or above **	1,536 (11.2)	1,505 (11.5)	31 (4.4)	<.001
**Smoked^b^**	3,953 (28.7)	3,689 (28.3)	264 (37.2)	<.001
**Drank alcohol**	1,745 (12.7)	1,676 (12.8)	69 (9.7)	.02
**Physical activity**				
Low	4,049 (29.4)	3,685 (28.2)	364 (51.3)	<.001
Moderate	2,940 (21.4)	2,834 (21.7)	106 (14.9)	
High	6,771 (49.2)	6,531 (50.1)	240 (33.8)	
**BMI (kg/m^2^), median (IQR)**	23.6 (21.4–26.1)	23.7 (21.4–26.1)	22.6 (20.8–25.2)	<.001
**Systolic blood pressure, mm Hg, median (IQR)**	119.0 (109.3–130.0)	118.7 (109.0–129.7)	126.3 (115.3–141.7)	<.001
**Diastolic blood pressure, mm Hg, median (IQR)**	75.3 (69.3–82.0)	75.3 (69.3–82.0)	76.3 (69.0–84.3)	.046
**Total cholesterol, mmol/L, median (IQR)**	4.3 (3.8–5.0)	4.3 (3.8–5.0)	4.5 (3.9–5.1)	.008
**Triglycerides, mmol/L, median (IQR)**	1.3 (0.9–1.9)	1.3 (0.9–1.9)	1.3 (0.9–1.8)	.08
**HDL cholesterol, mmol/L, median (IQR)**	1.1 (1.0–1.3)	1.1 (1.0–1.3)	1.1 (1.0–1.3)	.009
**LDL cholesterol, mmol/L, median (IQR)**	2.5 (2.0–3.0)	2.5 (2.0–3.0)	2.6 (2.2–3.1)	<.001
**Fasting plasma glucose, mmol/L, median (IQR)**	5.3 (5.0–5.7)	5.3 (5.0–5.7)	5.4 (5.0–6.0)	<.001

Abbreviations: BMI, body mass index; CNY, Chinese Yuan; HDL, high-density lipoprotein; IQR, interquartile range; LDL, low-density lipoprotein.

a Calculated at a mean follow-up of 5.95 years (81,856 person years). Values are number (percentage) unless otherwise indicated.

b Defined as currently smoking and/or having smoked 100 or more cigarettes during their lifetime.

**Figure 2:**
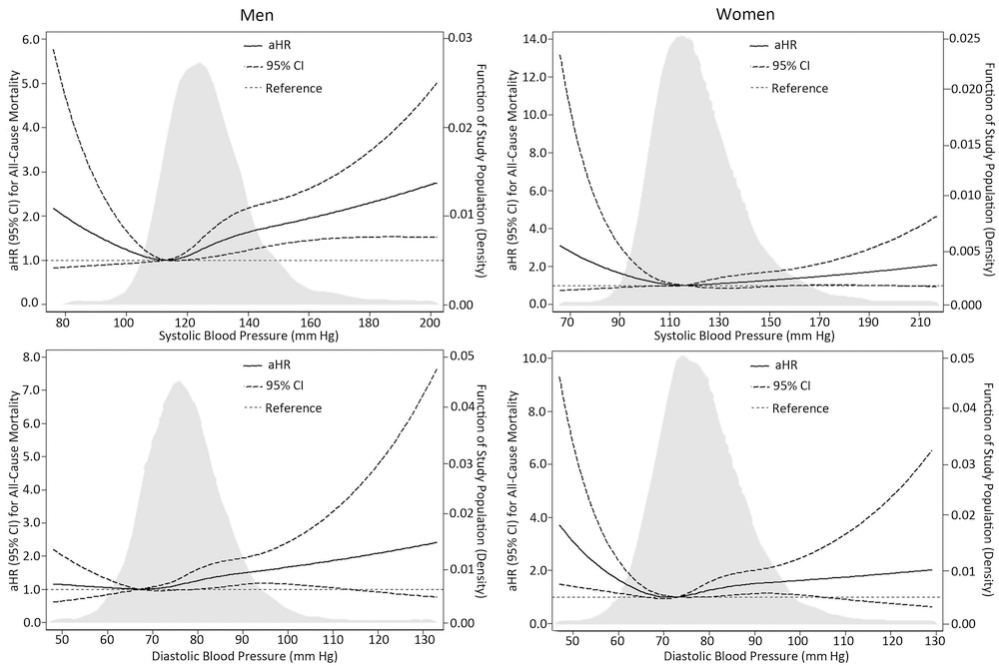
Adjusted risk trends for all-cause mortality by blood pressure level at baseline examination on a continuous scale for men and women, adjusted for age; marital status; mean individual income (monthly); education level; smoking; drinking; physical activity; body mass index; total cholesterol; triglycerides; high-density lipoprotein cholesterol; low-density lipoprotein cholesterol; fasting plasma glucose; family history of hypertension, diabetes mellitus, or hyperlipidemia; and use of hypoglycemic and lipid-lowering medications. Abbreviations: —, not applicable; aHR, adjusted hazard ratio; CI, confidence interval; DBP, diastolic blood pressure; SBP, systolic blood pressure.

**Figure 3:**
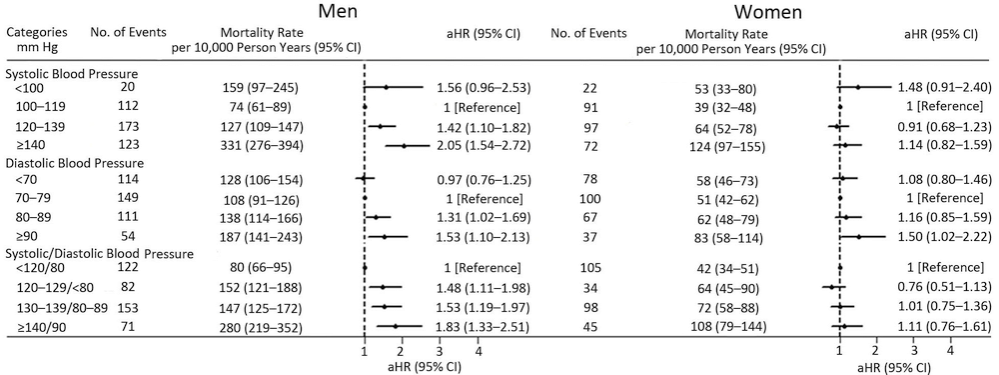
All-cause mortality risk per 1,000 person years by blood pressure categories at baseline examination in men and women based on the 2017 US hypertension guidelines (11), adjusted for age; marital status; mean individual income (monthly); education level; smoking; drinking; physical activity; body mass index; total cholesterol; triglycerides; high-density lipoprotein cholesterol; low-density lipoprotein cholesterol; fasting plasma glucose; family history of hypertension, diabetes mellitus, or hyperlipidemia; and use of hypoglycemic and lipid-lowering medications. Abbreviations: aHR, adjusted hazard ratio; CI, confidence interval.

**SBP–mortality relationship. **We observed a slight U-shaped relationship between SBP at baseline examination and all-cause mortality for men and women as a continuous variable after adjusting for some potential confounders, although the 95% CIs for some ranges of SBP or DBP were not significant. The lowest all-cause mortality was associated with SBP of 113 mm Hg for men and 116 mm Hg for women (Figure 2). For men, risk of all-cause mortality increased with SBP of 120–139 mm Hg (adjusted HR [aHR] 1.42; 95% CI, 1.10–1.82) or ≥140 mm Hg (aHR 2.05; 95% CI, 1.54–2.72) compared with SBP of 100 mm Hg to 119 mm Hg. We did not find any relationship between SBP categories and all-cause mortality for women (Figure 3). The results was consistent with Appendix Table 2, which also takes diabetes mellitus as the adjustment variable.

**Table:** 

Men				Women			
SBP, mm Hg	aHR (95% CI)	DBP, mm Hg	aHR (95% CI)	SBP, mm Hg	aHR (95% CI)	DBP, mm Hg	aHR (95% CI)
76	2.17 (0.82–5.77)	48	1.16 (0.61–2.20)	66	3.09 (0.73–13.17)	42	5.08 (1.64–15.76)
77	2.12 (0.82–5.48)	49	1.15 (0.63–2.11)	67	3.01 (0.73–12.40)	43	4.77 (1.61–14.18)
78	2.07 (0.83–5.20)	50	1.14 (0.64–2.02)	68	2.93 (0.74–11.66)	44	4.48 (1.58–12.76)
79	2.03 (0.83–4.94)	51	1.13 (0.66–1.93)	69	2.86 (0.74–10.97)	45	4.21 (1.55–11.48)
80	1.98 (0.84–4.69)	52	1.12 (0.68–1.85)	70	2.78 (0.75–10.33)	46	3.96 (1.52–10.33)
81	1.93 (0.84–4.45)	53	1.11 (0.70–1.77)	71	2.71 (0.76–9.72)	47	3.72 (1.49–9.29)
82	1.89 (0.84–4.22)	54	1.10 (0.72–1.69)	72	2.64 (0.76–9.14)	48	3.49 (1.46–8.36)
83	1.84 (0.85–4.01)	55	1.09 (0.73–1.62)	73	2.57 (0.77–8.60)	49	3.28 (1.43–7.52)
84	1.80 (0.85–3.81)	56	1.08 (0.75–1.55)	74	2.51 (0.78–8.10)	50	3.08 (1.40–6.77)
85	1.76 (0.86–3.62)	57	1.07 (0.77–1.49)	75	2.44 (0.78–7.62)	51	2.89 (1.37–6.09)
86	1.72 (0.86–3.43)	58	1.06 (0.79–1.42)	76	2.38 (0.79–7.17)	52	2.72 (1.35–5.48)
87	1.68 (0.86–3.26)	59	1.05 (0.82–1.36)	77	2.32 (0.80–6.75)	53	2.55 (1.32–4.93)
88	1.64 (0.87–3.10)	60	1.05 (0.84–1.31)	78	2.26 (0.80–6.35)	54	2.40 (1.29–4.44)
89	1.60 (0.87–2.94)	61	1.04 (0.86–1.25)	79	2.20 (0.81–5.97)	55	2.25 (1.27–4.00)
90	1.56 (0.88–2.79)	62	1.03 (0.88–1.20)	80	2.14 (0.81–5.62)	56	2.11 (1.24–3.60)
91	1.53 (0.88–2.65)	63	1.02 (0.91–1.15)	81	2.08 (0.82–5.29)	57	1.99 (1.22–3.24)
92	1.49 (0.89–2.52)	64	1.01 (0.93–1.10)	82	2.03 (0.83–4.98)	58	1.86 (1.19–2.92)
93	1.46 (0.89–2.39)	65	1.01 (0.95–1.06)	83	1.98 (0.83–4.69)	59	1.75 (1.17–2.63)
94	1.42 (0.89–2.27)	66	1.00 (0.98–1.03)	84	1.93 (0.84–4.41)	60	1.65 (1.14–2.37)
95	1.39 (0.90–2.15)	67	1.00 (1.00–1.00)	85	1.88 (0.85–4.15)	61	1.55 (1.12–2.13)
96	1.36 (0.90–2.04)	68	1.00 (0.98–1.02)	86	1.83 (0.86–3.91)	62	1.45 (1.09–1.93)
97	1.33 (0.91–1.94)	69	1.00 (0.96–1.05)	87	1.78 (0.86–3.68)	63	1.36 (1.07–1.74)
98	1.30 (0.91–1.84)	70	1.01 (0.95–1.07)	88	1.73 (0.87–3.46)	64	1.28 (1.04–1.58)
99	1.27 (0.92–1.75)	71	1.02 (0.95–1.10)	89	1.69 (0.88–3.26)	65	1.21 (1.02–1.44)
100	1.24 (0.92–1.66)	72	1.04 (0.95–1.13)	90	1.65 (0.88–3.07)	66	1.15 (0.99–1.32)
101	1.21 (0.92–1.58)	73	1.06 (0.96–1.16)	91	1.60 (0.89–2.89)	67	1.09 (0.97–1.23)
102	1.18 (0.93–1.50)	74	1.08 (0.96–1.21)	92	1.56 (0.90–2.72)	68	1.05 (0.95–1.16)
103	1.15 (0.93–1.42)	75	1.11 (0.97–1.26)	93	1.52 (0.90–2.56)	69	1.02 (0.94–1.10)
104	1.13 (0.94–1.36)	76	1.14 (0.97–1.32)	94	1.48 (0.91–2.41)	70	0.99 (0.93–1.06)
105	1.10 (0.94–1.29)	77	1.17 (0.98–1.39)	95	1.44 (0.92–2.27)	71	0.98 (0.94–1.03)
106	1.08 (0.95–1.23)	78	1.20 (0.98–1.47)	96	1.40 (0.92–2.14)	72	0.99 (0.96–1.01)
107	1.06 (0.95–1.18)	79	1.23 (0.99–1.54)	97	1.37 (0.93–2.02)	73	1.00 (1.00–1.00)
108	1.04 (0.96–1.14)	80	1.26 (1.00–1.60)	98	1.33 (0.93–1.90)	74	1.02 (0.99–1.05)
109	1.03 (0.96–1.10)	81	1.29 (1.01–1.66)	99	1.30 (0.94–1.79)	75	1.05 (0.99–1.12)
110	1.02 (0.97–1.06)	82	1.32 (1.02–1.71)	100	1.27 (0.95–1.69)	76	1.09 (0.98–1.20)
111	1.01 (0.98–1.04)	83	1.35 (1.03–1.76)	101	1.23 (0.95–1.60)	77	1.13 (0.98–1.29)
112	1.00 (0.99–1.02)	84	1.37 (1.05–1.79)	102	1.20 (0.95–1.52)	78	1.17 (0.99–1.39)
113	1.00 (1.00–1.00)	85	1.39 (1.06–1.82)	103	1.18 (0.96–1.45)	79	1.21 (0.99–1.48)
114	1.00 (0.99–1.01)	86	1.41 (1.08–1.85)	104	1.15 (0.96–1.38)	80	1.25 (1.00–1.57)
115	1.01 (0.98–1.03)	87	1.43 (1.10–1.87)	105	1.12 (0.96–1.32)	81	1.29 (1.01–1.65)
116	1.02 (0.98–1.05)	88	1.45 (1.11–1.90)	106	1.10 (0.96–1.27)	82	1.32 (1.02–1.71)
117	1.03 (0.99–1.08)	89	1.47 (1.13–1.92)	107	1.08 (0.96–1.22)	83	1.36 (1.04–1.77)
118	1.05 (0.99–1.12)	90	1.49 (1.14–1.94)	108	1.06 (0.95–1.18)	84	1.38 (1.05–1.82)
119	1.07 (1.00–1.16)	91	1.51 (1.16–1.97)	109	1.05 (0.95–1.15)	85	1.41 (1.06–1.87)
120	1.10 (1.00–1.20)	92	1.52 (1.16–2.00)	110	1.03 (0.95–1.12)	86	1.43 (1.08–1.90)
121	1.12 (1.01–1.25)	93	1.54 (1.17–2.03)	111	1.02 (0.95–1.10)	87	1.45 (1.09–1.93)
122	1.15 (1.02–1.31)	94	1.56 (1.17–2.07)	112	1.01 (0.95–1.08)	88	1.47 (1.10–1.96)
123	1.19 (1.03–1.37)	95	1.58 (1.18–2.12)	113	1.01 (0.96–1.06)	89	1.49 (1.11–1.98)
124	1.22 (1.03–1.43)	96	1.59 (1.17–2.17)	114	1.00 (0.97–1.04)	90	1.50 (1.12–2.01)
125	1.25 (1.04–1.50)	97	1.61 (1.17–2.22)	115	1.00 (0.98–1.02)	91	1.52 (1.13–2.03)
126	1.28 (1.05–1.56)	98	1.63 (1.17–2.28)	116	1.00 (1.00–1.00)	92	1.53 (1.13–2.06)
127	1.32 (1.06–1.63)	99	1.65 (1.16–2.34)	117	1.00 (0.98–1.02)	93	1.54 (1.13–2.09)
128	1.35 (1.07–1.69)	100	1.67 (1.15–2.41)	118	1.00 (0.97–1.05)	94	1.55 (1.13–2.13)
129	1.38 (1.08–1.75)	101	1.69 (1.14–2.49)	119	1.01 (0.95–1.07)	95	1.56 (1.12–2.17)
130	1.40 (1.10–1.80)	102	1.71 (1.13–2.56)	120	1.01 (0.93–1.10)	96	1.58 (1.12–2.22)
131	1.43 (1.11–1.85)	103	1.72 (1.12–2.65)	121	1.02 (0.92–1.13)	97	1.59 (1.11–2.28)
132	1.46 (1.12–1.90)	104	1.74 (1.11–2.73)	122	1.03 (0.91–1.17)	98	1.60 (1.10–2.33)
133	1.48 (1.13–1.95)	105	1.76 (1.10–2.82)	123	1.03 (0.89–1.20)	99	1.61 (1.08–2.40)
134	1.51 (1.14–1.99)	106	1.78 (1.09–2.92)	124	1.04 (0.88–1.23)	100	1.62 (1.07–2.46)
135	1.53 (1.16–2.03)	107	1.80 (1.08–3.02)	125	1.05 (0.87–1.26)	101	1.64 (1.05–2.54)
136	1.55 (1.17–2.06)	108	1.82 (1.07–3.12)	126	1.06 (0.87–1.29)	102	1.65 (1.04–2.61)
137	1.57 (1.18–2.09)	109	1.84 (1.05–3.23)	127	1.07 (0.86–1.32)	103	1.66 (1.02–2.69)
138	1.59 (1.20–2.12)	110	1.87 (1.04–3.34)	128	1.07 (0.85–1.35)	104	1.67 (1.01–2.78)
139	1.61 (1.21–2.15)	111	1.89 (1.03–3.46)	129	1.08 (0.85–1.38)	105	1.69 (0.99–2.86)
140	1.63 (1.22–2.18)	112	1.91 (1.01–3.58)	130	1.09 (0.85–1.40)	106	1.70 (0.98–2.96)
141	1.65 (1.24–2.20)	113	1.93 (1.00–3.71)	131	1.10 (0.85–1.42)	107	1.71 (0.96–3.05)
142	1.67 (1.25–2.22)	114	1.95 (0.99–3.85)	132	1.11 (0.85–1.45)	108	1.72 (0.94–3.16)
143	1.68 (1.27–2.24)	115	1.97 (0.98–3.98)	133	1.11 (0.85–1.47)	109	1.74 (0.93–3.26)
144	1.70 (1.28–2.26)	116	1.99 (0.96–4.13)	134	1.12 (0.85–1.49)	110	1.75 (0.91–3.37)
145	1.72 (1.29–2.28)	117	2.02 (0.95–4.28)	135	1.13 (0.85–1.51)	111	1.76 (0.89–3.49)
146	1.73 (1.31–2.30)	118	2.04 (0.94–4.43)	136	1.14 (0.85–1.52)	112	1.78 (0.88–3.61)
147	1.75 (1.32–2.31)	119	2.06 (0.93–4.60)	137	1.15 (0.86–1.54)	113	1.79 (0.86–3.73)
148	1.76 (1.33–2.33)	120	2.09 (0.91–4.77)	138	1.16 (0.86–1.55)	114	1.80 (0.84–3.86)
149	1.78 (1.34–2.35)	121	2.11 (0.90–4.94)	139	1.16 (0.86–1.57)	115	1.82 (0.83–3.99)
150	1.79 (1.35–2.37)	122	2.13 (0.89–5.12)	140	1.17 (0.87–1.58)	116	1.83 (0.81–4.13)
151	1.81 (1.37–2.39)	123	2.16 (0.88–5.31)	141	1.18 (0.88–1.60)	117	1.85 (0.80–4.28)
152	1.82 (1.38–2.41)	124	2.18 (0.86–5.51)	142	1.19 (0.88–1.61)	118	1.86 (0.78–4.43)
153	1.84 (1.39–2.43)	125	2.21 (0.85–5.71)	143	1.20 (0.89–1.62)	119	1.87 (0.76–4.59)
154	1.85 (1.40–2.45)	126	2.23 (0.84–5.92)	144	1.21 (0.89–1.63)	120	1.89 (0.75–4.75)
155	1.87 (1.41–2.48)	127	2.26 (0.83–6.14)	145	1.22 (0.90–1.65)	121	1.90 (0.73–4.92)
156	1.88 (1.42–2.50)	128	2.28 (0.82–6.37)	146	1.23 (0.91–1.66)	122	1.92 (0.72–5.10)
157	1.90 (1.42–2.53)	129	2.31 (0.81–6.61)	147	1.24 (0.91–1.67)	123	1.93 (0.71–5.28)
158	1.91 (1.43–2.56)	130	2.33 (0.79–6.86)	148	1.25 (0.92–1.68)	124	1.94 (0.69–5.47)
159	1.93 (1.44–2.58)	131	2.36 (0.78–7.11)	149	1.25 (0.93–1.70)	125	1.96 (0.68–5.66)
160	1.94 (1.45–2.61)	132	2.39 (0.77–7.38)	150	1.26 (0.94–1.71)	126	1.97 (0.66–5.87)
161	1.96 (1.45–2.65)	133	2.41 (0.76–7.65)	151	1.27 (0.94–1.72)	127	1.99 (0.65–6.08)
162	1.98 (1.46–2.68)	—	—	152	1.28 (0.95–1.74)	128	2.00 (0.64–6.30)
163	1.99 (1.47–2.71)	—	—	153	1.29 (0.95–1.75)	129	2.02 (0.63–6.52)
164	2.01 (1.47–2.75)	—	—	154	1.30 (0.96–1.77)	—	—
165	2.03 (1.48–2.78)	—	—	155	1.31 (0.96–1.79)	—	—
166	2.04 (1.48–2.82)	—	—	156	1.32 (0.97–1.80)	—	—
167	2.06 (1.49–2.86)	—	—	157	1.33 (0.97–1.82)	—	—
168	2.08 (1.49–2.90)	—	—	158	1.34 (0.98–1.84)	—	—
169	2.10 (1.49–2.94)	—	—	159	1.35 (0.98–1.86)	—	—
170	2.11 (1.50–2.98)	—	—	160	1.36 (0.99–1.88)	—	—
171	2.13 (1.50–3.02)	—	—	161	1.37 (0.99–1.90)	—	—
172	2.15 (1.50–3.07)	—	—	162	1.38 (0.99–1.93)	—	—
173	2.17 (1.51–3.11)	—	—	163	1.39 (0.99–1.95)	—	—
174	2.18 (1.51–3.16)	—	—	164	1.40 (1.00–1.98)	—	—
175	2.20 (1.51–3.21)	—	—	165	1.41 (1.00–2.00)	—	—
176	2.22 (1.51–3.26)	—	—	166	1.42 (1.00–2.03)	—	—
177	2.24 (1.52–3.31)	—	—	167	1.44 (1.00–2.05)	—	—
178	2.26 (1.52–3.36)	—	—	168	1.45 (1.00–2.08)	—	—
179	2.28 (1.52–3.41)	—	—	169	1.46 (1.01–2.11)	—	—
180	2.29 (1.52–3.46)	—	—	170	1.47 (1.01–2.14)	—	—
181	2.31 (1.52–3.52)	—	—	171	1.48 (1.01–2.17)	—	—
182	2.33 (1.52–3.58)	—	—	172	1.49 (1.01–2.20)	—	—
183	2.35 (1.52–3.63)	—	—	173	1.50 (1.01–2.24)	—	—
184	2.37 (1.52–3.69)	—	—	174	1.51 (1.01–2.27)	—	—
185	2.39 (1.52–3.75)	—	—	175	1.52 (1.01–2.30)	—	—
186	2.41 (1.52–3.82)	—	—	176	1.54 (1.01–2.34)	—	—
187	2.43 (1.52–3.88)	—	—	177	1.55 (1.01–2.37)	—	—
188	2.45 (1.52–3.94)	—	—	178	1.56 (1.01–2.41)	—	—
189	2.47 (1.52–4.01)	—	—	179	1.57 (1.01–2.45)	—	—
190	2.49 (1.52–4.08)	—	—	180	1.58 (1.01–2.49)	—	—
191	2.51 (1.52–4.15)	—	—	181	1.59 (1.01–2.53)	—	—
192	2.53 (1.52–4.22)	—	—	182	1.61 (1.00–2.57)	—	—
193	2.55 (1.52–4.29)	—	—	183	1.62 (1.00–2.61)	—	—
194	2.58 (1.52–4.36)	—	—	184	1.63 (1.00–2.65)	—	—
195	2.60 (1.52–4.44)	—	—	185	1.64 (1.00–2.69)	—	—
196	2.62 (1.52–4.51)	—	—	186	1.65 (1.00–2.74)	—	—
197	2.64 (1.52–4.59)	—	—	187	1.67 (1.00–2.78)	—	—
198	2.66 (1.52–4.67)	—	—	188	1.68 (1.00–2.83)	—	—
199	2.68 (1.52–4.75)	—	—	189	1.69 (1.00–2.87)	—	—
200	2.71 (1.52–4.83)	—	—	190	1.70 (0.99–2.92)	—	—
201	2.73 (1.51–4.92)	—	—	191	1.72 (0.99–2.97)	—	—
202	2.75 (1.51–5.00)	—	—	192	1.73 (0.99–3.02)	—	—
—	—	—	—	193	1.74 (0.99–3.07)	—	—
—	—	—	—	194	1.76 (0.99–3.12)	—	—
—	—	—	—	195	1.77 (0.98–3.18)	—	—
—	—	—	—	196	1.78 (0.98–3.23)	—	—
—	—	—	—	197	1.80 (0.98–3.29)	—	—
—	—	—	—	198	1.81 (0.98–3.34)	—	—
—	—	—	—	199	1.82 (0.98–3.40)	—	—
—	—	—	—	200	1.84 (0.97–3.46)	—	—
—	—	—	—	201	1.85 (0.97–3.52)	—	—
—	—	—	—	202	1.86 (0.97–3.58)	—	—
—	—	—	—	203	1.88 (0.97–3.64)	—	—
—	—	—	—	204	1.89 (0.97–3.71)	—	—
—	—	—	—	205	1.91 (0.96–3.77)	—	—
—	—	—	—	206	1.92 (0.96–3.84)	—	—
—	—	—	—	207	1.93 (0.96–3.90)	—	—
—	—	—	—	208	1.95 (0.96–3.97)	—	—
—	—	—	—	209	1.96 (0.95–4.04)	—	—
—	—	—	—	210	1.98 (0.95–4.11)	—	—
—	—	—	—	211	1.99 (0.95–4.19)	—	—
—	—	—	—	212	2.01 (0.95–4.26)	—	—
—	—	—	—	213	2.02 (0.94–4.34)	—	—
—	—	—	—	214	2.04 (0.94–4.41)	—	—
—	—	—	—	215	2.05 (0.94–4.49)	—	—
—	—	—	—	216	2.07 (0.94–4.57)	—	—
—	—	—	—	217	2.08 (0.93–4.65)	—	—

**Table:** 

Category	Men			Women		
	Event	Mortality rate (95% CI)	aHR (95% CI)	Event	Mortality rate (95% CI)	aHR (95% CI)
Systolic blood pressure, mmHg						
<100	20	159 (97–245)	1.56 (0.96–2.53)	22	53 (33–80)	1.48 (0.91–2.40)
100–119	112	74 (61–89)	Ref	91	39 (32–48)	Ref
120–139	173	127 (109–147)	1.42 (1.10–1.82)	97	64 (52–78)	0.91 (0.68–1.23)
≥140	123	331 (276–394)	2.05 (1.54–2.72)	72	124 (97–155)	1.14 (0.82–1.59)
Diastolic blood pressure, mmHg						
<70	114	128 (106–154)	0.97 (0.76–1.25)	78	58 (46–73)	1.08 (0.80–1.46)
70–79	149	108 (91–126)	1 [Reference]	100	51 (42–62)	1 [Reference]
80–89	111	138 (114–243)	1.31 (1.02–1.69)	67	62 (48–79)	1.16 (0.85–1.59)
≥90	54	187 (141–243)	1.53 (1.10–2.13)	37	83 (58–114)	1.50 (1.02–2.22)
Systolic blood pressure/diastolic blood pressure, mmHg						
<120/80	122	80 (66–95)	1 [Reference]	105	42 (34–51)	1 [Reference]
120–129/< 80	82	152 (121–188)	1.48 (1.11–1.98)	34	64 (45–90)	0.76 (0.51–1.13)
130–139/80–89	153	147 (125–172)	1.53 (1.19–1.97)	98	72 (58–88)	1.01 (0.75–1.36)
≥140/90	71	280 (219–352)	1.83 (1.33–2.51)	45	108 (79–144)	1.11 (0.76–1.61)

**Figure 4:**
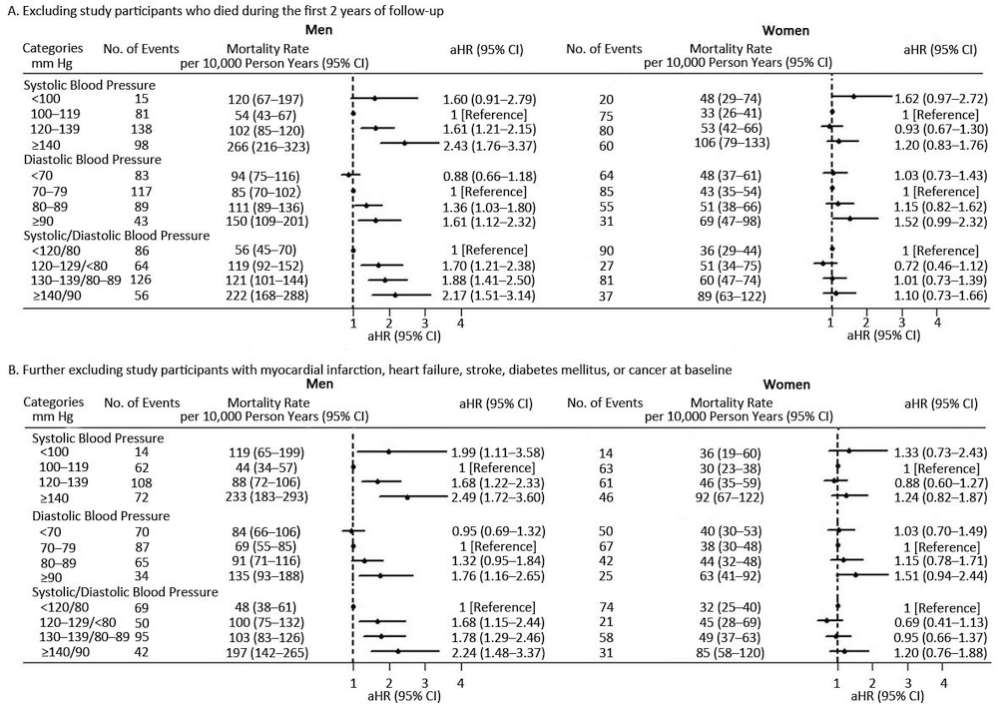
All-cause mortality rate per 1,000 person years by blood pressure categories based on the 2017 US hypertension guidelines (11) at baseline examination for men and women on sensitivity analyses, adjusted for age; marital status; mean individual income (monthly); education level; smoking; drinking; physical activity; body mass index; total cholesterol; triglycerides; high-density lipoprotein cholesterol; low-density lipoprotein cholesterol; fasting plasma glucose; family history of hypertension, diabetes mellitus, or hyperlipidemia; and use of hypoglycemic and lipid-lowering medications. Abbreviations: aHR, adjusted hazard ratio; CI, confidence interval.

The results did not change on sensitivity analyses after excluding deaths occurring during the first 2 years of follow-up. On restricting the analyses to participants without pre-existing chronic disease (cardiovascular disease, diabetes mellitus, or cancer), results were consistent with the initial analyses (Figure 3) except for a significant association for men with SBP less than 100 mm Hg (aHR 1.99; 95% CI, 1.11–3.58) (Figure 4). Further taking diabetes mellitus as the adjustment variable, the results did not change (Appendix Table 2).

**Table:** 

Category	Men				Women		
	No. of Events	Mortality Rate per 10,000 Person Years (95% CI)	aHR (95% CI)		No. of Events	Mortality Rate per 10,000 Person Years (95% CI)	aHR (95% CI)
Excluding Study Participants Who Died During First 2 Years of Follow-Up							
Systolic blood pressure, mmHg							
<100	15	120 (67–197)	1.60 (0.91–2.79)	20		48 (29–74)	1.62 (0.97–2.72)
100-119	81	54 (43–67)	1 [Reference]	75		33 (26–41)	1 [Reference]
120-139	138	102 (85–120)	1.61 (1.21–2.15)	80		53 (42–66)	0.93 (0.67–1.30)
≥140	98	266 (216–323)	2.43 (1.76–3.37)	60		103 (79–133)	1.20 (0.83–1.76)
Diastolic blood pressure, mHg							
<70	83	94 (75–116)	0.88 (0.66–1.18)	64		48 (37–61)	1.03 (0.73–1.43)
70-79	117	85 (70–102)	1 [Reference]	85		43 (35–54)	1 [Reference]
80-89	89	111 (89–136)	1.36 (1.03–1.80)	55		51 (38–66)	1.15 (0.82–1.62)
≥90	43	150 (109–201)	1.61 (1.12–2.32)	31		69 (47–98)	1.52 (0.99–2.32)
Systolic/diastolic blood pressure, mmHg							
<120/80	86	56 (45–70)	1 [Reference]	90		36 (29–44)	1 [Reference]
120-129/< 80	64	119 (92–152)	1.70 (1.21–2.38)	27		51 (34–75)	0.72 (0.46–1.12)
130-139/80-89	126	121 (101–144)	1.88 (1.41–2.50)	81		60 (47–74)	1.01 (0.73–1.39)
≥140/90	56	222 (168–288)	2.17 (1.51–3.14)	37		89 (63–122)	1.10 (0.73–1.66)
Further Excluding Study Participants With Myocardia Infarction, Heart Failure, Stroke, Diabetes Mellitus, or Cancer at Baseline							
Systolic blood pressure, mmHg							
<100	14	119 (65–199)	1.99 (1.11–3.58)	14		36 (19–60)	1.33 (0.73–2.43)
100-119	62	44 (34–57)	1 [Reference]	63		30 (23–38)	1 [Reference]
120-139	108	88 (72–106)	1.68 (1.22–2.33)	61		46 (35–59)	0.88 (0.60–1.27)
≥140	72	233 (183–293)	2.49 (1.72–3.60)	46		92 (67–122)	1.24 (0.82–1.87)
Diastolic blood pressure, mmHg							
<70	70	84 (66–106)	0.95 (0.69–1.32)	50		40 (30–53)	1.03 (0.70–1.49)
70-79	87	69 (55–85)	1 [Reference]	67		38 (30–48)	1 [Reference]
80-89	65	91 (71–116)	1.32 (0.95–1.84)	42		44 (32–48)	1.15 (0.78–1.71)
≥90	34	135 (93–188)	1.76 (1.16–2.65)	25		63 (41–92)	1.51 (0.94–2.44)
Systolic/diastolic blood pressure, mmHg							
<120/80	69	48 (38–61)	1 [Reference]	74		32 (25–40)	1 [Reference]
120-129/<80	50	100 (75–132)	1.68 (1.15–2.44)	21		45 (28–69)	0.69 (0.41–1.13)
130-139/80-89	95	103 (83–126)	1.78 (1.29–2.46)	58		49 (37–63)	0.95 (0.66–1.37)
≥140/90	42	197 (142–265)	2.24 (1.48–3.37)	31		85 (58–120)	1.20 (0.76–1.88)

**DBP–mortality relationship. **We assessed the DBP–mortality relationship by using continuous DBP at baseline and found a slight U shape for both sexes, although the 95% CIs for some ranges of SBP or DBP were not significant. The DBP levels associated with the lowest all-cause mortality were 67 mm Hg for men and 73 mm Hg for women (Figure 3). All-cause mortality was positively associated with DBP of 80 mm Hg to 89 mm Hg (aHR 1.31; 95% CI, 1.02–1.69) or DBP ≥90 mm Hg (aHR 1.53; 95% CI, 1.10–2.13) for men and DBP ≥90 mm Hg (aHR 1.50; 95% CI, 1.02–2.22) for women compared with people with DBP 70 mm Hg to 79 mm Hg (Figure 3). The results, further taking diabetes mellitus as the adjustment variable, were consistent with Appendix Table 2.

When we excluded study participants who died during the first 2 years of follow-up, we saw no change in the relationship between DBP and mortality for men: however, we saw a marginal association for women with DBP ≥90 mm Hg (aHR 1.52; 95% CI, 0.99–2.32) (Figure 4A). When we further restricted analysis to people free of the 5 pre-existing chronic diseases at baseline, results were mostly similar to the initial analyses (Figure 3) for men with DBP ≥90 mm Hg (aHR 1.76; 95% CI, 1.16–2.65); the relationship between DPB and mortality further weakened for women with DBP at or above 90 mm Hg (aHR 1.51; 95% CI, 0.94–2.44) (Figure 4B). However, the association between DBP and mortality disappeared for men with DBP 80 to 89 mm Hg (aHR 1.32; 95% CI, 0.95–1.84). Further taking diabetes mellitus as the adjustment variable, the results did not change (Appendix Table 2).

**Association of all-cause mortality with blood pressure categories based on the 2017 US hypertension guidelines. **For men, the risk of all-cause mortality was higher and increased with increasing blood pressure according to 2017 US hypertension guidelines: with elevated blood pressure (aHR 1.48; 95% CI, 1.11–1.98), with stage 1 hypertension (aHR 1.53; 95% CI, 1.19–1.97), or with stage 2 hypertension (aHR 1.83; 95% CI, 1.33–2.51) compared with normal blood pressure. However, we found no significant relationship for women between blood pressure categories and all-cause mortality (Figure 3). The results were consistent with Appendix Table 2, which also takes diabetes mellitus as the adjustment variable.

When we excluded deaths that occurred during the first 2 years of follow-up, elevated blood pressure (aHR 1.70; 95% CI, 1.21–2.38), stage 1 hypertension (aHR 1.88; 95% CI, 1.41–2.50), or stage 2 hypertension (aHR 2.17; 95% CI 1.51–3.14) were positively associated with all-cause mortality for men but not women compared with normal blood pressure (Figure 4A), with increased strength as compared with our initial analyses (Figure 3). On further restricting the analysis to participants without any of the 5 pre-existing chronic diseases, risk of all-cause mortality was increased for men but not women with blood pressure status as defined by 2017 US hypertension guidelines as elevated blood pressure (aHR 1.68; 95% CI, 1.15–2.44), stage 1 hypertension (aHR 1.78; 95% CI, 1.29–2.46), or stage 2 hypertension (aHR 2.24; 95% CI 1.48–3.37) compared with normal blood pressure (Figure 4B). Further taking diabetes mellitus as the adjustment variable, the results did not change (Appendix Table 2).

## Discussion

In this prospective cohort study of 13,760 eligible rural Chinese adults, results suggested a U-shaped association of SBP or DBP with all-cause mortality for both sexes. Men with SBP ≥120 mm Hg, DBP ≥90 mm Hg, or SBP/DBP ≥130/80 mm Hg showed increased risk of all-cause mortality. However, we found no significant relationship between SBP, DBP, or the 2017 US hypertension guidelines categories and all-cause mortality for women.

Several cohort studies indicated a positive association of increased mortality risk with low SBP level (<120 mm Hg/90 mm Hg) or low DBP level (<80 mm Hg/40 mm Hg): the Korean Cancer Prevention Study, with 22.7 million person years of follow-up, which included 1,329,525 people aged 30 to 95 (2); a retrospective community-based cohort study of 128,765 Taiwanese people aged 65 or older followed for 3 years (4); and a prospective cohort study of 4,658 Chinese people aged 65 to 99 followed for 3 years (8). Other cohort studies suggested increased mortality risk with high SBP level (≥120 mm Hg/160 mm Hg), high DBP level (≥90 mm Hg), or SBP/DBP ≥120/80 mm Hg, 140/90 mm Hg, 160/100 mm Hg (2,5,6,9,26,27).

Sex differences were observed in published cohort studies (2,6,7,28,29). Research data based on a cohort study from Korea indicated high mortality risk for men with SBP ≥140 mm Hg or <90 mm Hg and women with SBP ≥120 mm Hg (2). A Japanese cohort study with 11-year follow-up included 33,372 men and women aged 40 to 69 who had no prior diagnosis of cancer or cardiovascular disease. That study suggested increased mortality risk for men with SBP/DBP ≥130/85 mm Hg but not for women (28). However, a Swedish cohort study with 26-year follow-up that included 2,280 people aged 18 to 65 found the reverse results (increased mortality risk for women with SBP/DBP ≥130/85 mm Hg but not for men) (29). Evidence from Chinese cohort studies (6,7) indicated high mortality risk for men with SBP <100 mm Hg/120 mm Hg. For women, these studies showed high mortality risk with SBP ≥140 mm Hg/160 mm Hg, DBP ≥90 mm Hg, and SBP/DBP 140/90 mm Hg or even 160/100 mm Hg. Participants in these Chinese studies were coal miners and urban women.

Our study suggested an association of all-cause mortality for men with high SBP (≥120 mm Hg) and high DBP (≥90 mm Hg), or SBP/DBP ≥130/80 mm Hg, but not for women. The possible explanations for inconsistencies between our results and previous studies may be differences in socioeconomic status, demographic and physiologic characteristics, genetic predisposition, lifestyles, and environmental factors in multi-ethnic populations (11,30). Also, excessive lowering of blood pressure with the use of antihypertension medications would causally increase the risk of all-cause mortality (31), but previous studies ignored the effect of antihypertension medication apart from regarding it as a potential confounder. Only 1 study excluded people receiving antihypertension agents, but participants in that study were mainly young men aged 18 to 39 (26).

Our study showed a difference by sex in the blood pressure–mortality relationship. One explanation is that men could have greater psychological distress associated with mortality than women, a phenomenon more prevalent in poor regions, as in our rural study, than in urban areas (32,33). Also, the interaction between sex and genetic factors could be responsible for the different blood pressure–mortality association in men and women (34). Additionally, T cells play an important role in the pathogenesis of hypertension and are associated with a sex difference (35). High blood pressure is a major cause of cardiovascular disease mortality and a major contributor to all-cause mortality (36). Low blood pressure is associated with decreased myocardial perfusion pressure and myocardial ischemia (37). In addition, low blood pressure increases arterial stiffness, which increases mortality risk (38). The explanation for the association between blood pressure and risk of all-cause mortality is not intuitive, and the relevant mechanism should be further explored (36). Another possible reason may be different physiologic factors (39). However, we have no evidence of the relative benefits and harms of lowering blood pressure to recommended targets varying as a function of sex (40), and treatment guidelines are consistent for men and women (11,12).

Our study results suggest an association of blood pressure according to the 2017 US hypertension guidelines with all-cause mortality in rural Chinese adults and provide a useful reference for future studies. Lowering blood pressure to 130/80 mm Hg could be achieved by early hypertension screening and intervention (including active intervention in lifestyle), and the benefit may be greater among Asians than Westerners (30). The hypertension prevalence among rural Chinese in our study was 3 times higher according to the 2017 US hypertension guidelines (47.1% for men and 46.1% for women) than according to the 2018 Chinese hypertension guidelines (14.5% for men and 15.4% for women). The number of antihypertension treatments cannot increase significantly, and nonpharmacologic interventions, especially reducing salt intake and body weight, still remain a fundamentally important approach to hypertension control (11,30). Treatment could rely on clinicians’ cautious judgment about whether and when to start antihypertension treatment. In addition, setting goals for high blood pressure prevention and control may need to be tailored to people and populations with differing characteristics (27). Moreover, the awareness, treatment, and control rates of hypertension are low in China; a transition from knowing the problem to implementing the solution and improving management of medical therapy for hypertension is needed (30,41,42).

The primary strength of our study is its being the first to our knowledge to evaluate the association between the 2017 US hypertension guidelines and all-cause mortality in a Chinese population. Its second strength is its prospective design based on a general population with 81,856 person years of follow-up and the standardized longitudinal assessment of blood pressure and well-measured covariates. Finally, we assessed the sex-specific blood pressure–mortality relationship, whereas we previously had little evidence on the association of blood pressure categories with all-cause mortality by sex in China.

Nevertheless, our study has limitations. First, the generalizability of our findings to urban or to other ethnic populations than those in our study may be limited because study participants were rural Chinese adults. Second, despite sensitivity analyses of participants who had none of the 5 pre-existing chronic diseases, we could not completely exclude people with all chronic medical conditions. Finally, the possibility of residual confounding bias may remain because some covariates, such as psychological factors, were not investigated. Further studies are needed to evaluate sex differences in lowering blood pressure to an optimal level. Moreover, blood pressure–attributable mortality is a major public health challenge, and the combined efforts of health-policy makers, health care providers, and the general population are required to reduce the mortality burden caused by elevated blood pressure and hypertension.

## Appendix

**Appendix:** Table 1. Baseline Characteristics of People Included and Excluded, the Rural Chinese Cohort Study, 2007–2014

Variables	Included, n = 13,760	Excluded, n = 6,434	*P* Value
**Men**	5,661 (41.1)	2,272 (35.3)	<.001
**Age, y, median (IQR)**	49.0 (40.0–59.0)	55.0 (44.0–64.0)	<.001
**Married/cohabitating**	12,460 (90.6)	5,436 (84.5)	<.001
**Mean individual monthly income**			
<500 CNY	12,792 (93.2)	5,889 (91.9)	.003
500–1,000 CNY	744 (5.4)	420 (6.6)	
≥1,000 CNY	191 (1.4)	102 (1.6)	
**High school diploma or above**	1,536 (11.2)	722 (11.2)	.90
**Smoking**	3,953 (28.7)	1,429 (22.2)	<.001
**Drinking**	1,745 (12.7)	556 (8.6)	<.001
**Physical activity**			
Low	4,049 (29.4)	2,812 (43.7)	<.001
Moderate	2,940 (21.4)	1,339 (20.8)	
High	6,771 (49.2)	2,283 (35.5)	
**BMI, kg/m^2^, median (IQR)**	23.6 (21.4–26.1)	24.7 (22.2–27.5)	<.001
**SBP, mm Hg, median (IQR)**	119.0 (109.3–130.0)	134.3 (118.0–152.3)	<.001
**DBP, mm Hg, median (IQR)**	75.3 (69.3–82.0)	82.7 (74.0–92.0)	<.001
**Total cholesterol, mmol/L, median (IQR)**	4.3 (3.8–5.0)	4.5 (3.9–5.2)	<.001
**Triglycerides, mmol/L, median (IQR)**	1.3 (0.9–1.9)	1.5 (1.0–2.2)	<.001
**HDL-C, mmol/L, median (IQR)**	1.1 (1.0–1.3)	1.1 (1.0–1.3)	.002
**LDL-C, mmol/L, median (IQR)**	2.5 (2.0–3.0)	2.6 (2.1–3.1)	<.001
**Fasting plasma glucose, mmol/L, median (IQR)**	5.3 (5.0–5.7)	5.4 (5.1–5.9)	<.001

Abbreviations: BMI, body mass index; CNY, Chinese Yuan; DBP, diastolic blood pressure; FPG, fasting plasma glucose; HDL-C, high-density lipoprotein cholesterol; LDL-C, low-density lipoprotein cholesterol; IQR, interquartile range; SBP, systolic blood pressure.

**Appendix:** Table 2. All-Cause Mortality Risk by Blood Pressure Categories at Baseline Examination for Men And Women, the Rural Chinese Cohort Study, 2007–2014^a^

Categories	Men	Women
	aHR (95% CI)	aHR (95% CI)
**Systolic blood pressure, mmHg**		
<100	1.51 (0.93–2.45)	1.46 (0.90–2.38)
100–119	1 [Reference]	1 [Reference]
120–139	1.41 (1.10–1.80)	0.91 (0.67–1.23)
≥140	2.00 (1.51–2.66)	1.15 (0.82–1.60)
**Diastolic blood pressure, mmHg**		
<70	0.98 (0.76–1.26)	1.07 (0.79–1.46)
70–79	1 [Reference]	1 [Reference]
80–89	1.30 (1.01–1.67)	1.15 (0.84–1.58)
≥90	1.57 (1.13–2.18)	1.50 (1.02–2.21)
**Systolic blood pressure/diastolic blood pressure, mmHg**		
<120/80	1.80 (1.31–2.48)	1.12 (0.77–1.62)
120–129/<80	1 [Reference]	1 {Reference]
130–139/80–89	1.49 (1.11–1.99)	0.75 (0.51–1.12)
≥140/90	1.52 (1.18–1.95)	1.01 (0.75–1.36)

Abbreviations: aHR, adjusted hazard ratio; CI, confidence interval.

^a^ Adjusted for age, marital status, mean individual income (monthly), education level, smoking, drinking, physical activity, body mass index, total cholesterol, triglycerides, high-density lipoprotein cholesterol, low-density lipoprotein cholesterol, fasting plasma glucose, diabetes mellitus, family history of diseases (hypertension, diabetes mellitus, and hyperlipidemia), and use of medications (hypoglycemic and lipid-lowering agents).

**Appendix:** Table 3. All-Cause Mortality Risk by Blood Pressure Categories at Baseline Examination for Men and Women on Sensitivity Analyses, the Rural Chinese Cohort Study, 2007–2014^a^

Category	Men	Women
	aHR (95% CI)	aHR (95% CI)
**Systolic blood pressure, mmHg**		
<100	1.90 (1.08–3.35)	1.68 (1.00–2.83)
100–119	1 [Reference]	1 [Reference]
120–139	1.71 (1.25–2.32)	0.92 (0.65–1.30)
≥140	2.55 (1.80–3.62)	1.17 (0.80–1.72)
**Diastolic blood pressure, mmHg**		
<70	0.90 (0.66–1.22)	1.05 (0.74–1.48)
70–79	1 [Reference]	1 [Reference]
80–89	1.29 (0.95–1.75)	1.07 (0.74–1.54)
≥90	1.72 (1.16–2.54)	1.55 (1.00–2.39)
**Systolic blood pressure/diastolic blood pressure, mmHg**		
<120/80	2.29 (1.55–3.38)	1.09 (0.71–1.67)
120–129/<80	1 [Reference]	1 [Reference]
130–139/80–89	1.73 (1.21–2.46)	0.69 (0.43–1.09)
≥140/90	1.84 (1.35–2.50)	0.94 (0.67–1.31)

Abbreviations: aHR, adjusted hazard ratio; CI, confidence interval.

^a^ Sensitivity analyses excluding study participants who died during the first 2 years of follow-up and who had chronic disease at baseline examination (myocardial infarction, heart failure, stroke, or cancer. Adjusted for age, marital status, mean individual income (monthly), education level, smoking, drinking, physical activity, body mass index, total cholesterol, triglycerides, high-density lipoprotein cholesterol, low-density lipoprotein cholesterol, fasting plasma glucose, diabetes mellitus, family history of diseases (hypertension, diabetes mellitus, and hyperlipidemia), and use of medications (hypoglycemic and lipid-lowering agents).
